# VEGF-B inhibits hyperglycemia- and Macugen-induced retinal apoptosis

**DOI:** 10.1038/srep26059

**Published:** 2016-05-18

**Authors:** Delong Huang, Chen Zhao, Rong Ju, Anil Kumar, Geng Tian, Lijuan Huang, Lei Zheng, Xianglin Li, Lixian Liu, Shasha Wang, Xiangrong Ren, Zhimin Ye, Wei Chen, Liying Xing, Qishan Chen, Zhiqin Gao, Jia Mi, Zhongshu Tang, Bin Wang, Shuping Zhang, Chunsik Lee, Xuri Li

**Affiliations:** 1 Center for Medical and Pharmaceutical Research, Binzhou Medical University, Yantai, Shandong, 264003, P. R. China; 2 State Key Laboratory of Ophthalmology, Zhongshan Ophthalmic Center, Sun Yat-sen University, Guangzhou 510060, P. R. China; 3 Department of Ophthalmology, The First Affiliated Hospital of Nanjing Medical University and State Key Laboratory of Reproductive Medicine, Nanjing Medical University, Nanjing 210029, P. R. China; 4 Medical Imaging Institute, Shandong Province Characteristical Key Subject, Medical Imaging and Nuclear Medicine, Binzhou Medical University, Yantai, 264003 P. R. China; 5 Department of Cell Biology, Weifang Medical University, Weifang, 261053 P. R. China

## Abstract

Vascular endothelial growth factor B (VEGF-B) was discovered a long time ago. However, its role in hyperglycemia- and VEGF-A inhibition-induced retinal apoptosis remains unknown thus far. Yet, drugs that can block VEGF-B are being used to treat patients with diabetic retinopathy and other ocular neovascular diseases. It is therefore urgent to have a better understanding of the function of VEGF-B in these pathologies. Here, we report that both streptozotocin (STZ)-induced diabetes in rats and Macugen intravitreal injection in mice leads to retinal apoptosis in retinal ganglion cell and outer nuclear layers respectively. Importantly, VEGF-B treatment by intravitreal injection markedly reduced retinal apoptosis in both models. We further reveal that VEGF-B and its receptors, vascular endothelial growth factor 1 (VEGFR1) and neuropilin 1 (NP1), are abundantly expressed in rat retinae and choroids and are upregulated by high glucose with concomitant activation of Akt and Erk. These data highlight an important function of VEGF-B in protecting retinal cells from apoptosis induced by hyperglycemia and VEGF-A inhibition. VEGF-B may therefore have a therapeutic potential in treating various retinal degenerative diseases, and modulation of VEGF-B activity in the eye needs careful consideration.

Diabetic retinopathy (DR) is the leading cause of blindness in developed countries. DR is a common complication of diabetes, the incidence of which is rapidly increasing worldwide^1^. Conventionally, DR has been considered as a microcirculatory disease of the retina. However, emerging evidence has shown that retinal degeneration by apoptosis is an early event in DR. In fact, neural apoptosis is one of the most important histological features of DR^2^. Indeed, diabetes related apoptosis was detected in retinal ganglion cells (RGCs)^1^, and RGC loss has been found in both diabetic patients with no microcirculatory defect^3^,^4^,^5^ and STZ-induced diabetic rats^6^. In addition to DR, retinal apoptosis is also a potentially blinding pathology of numerous other ocular diseases, such as age-related macular degeneration, glaucoma, retinitis pigmentosa, retinal angiomatous proliferation and macular telangiectasia^1^,^7^,^8^, for which there is no satisfying treatment currently.

Anti-VEGF-A drugs have been used in the clinic to treat patients with DR to inhibit neovessels and edema^9^,^10^,^11^. However, despite of the beneficial effect, anti-VEGF-A treatment has been reported to be associated with the development of geographic atrophy (GA), the degeneration of retinal pigment epithelium (RPE) followed by the death of retinal neuronal cells. Indeed, it has been shown that within two years of anti-VEGF-A treatment, approximately 20–72% of patients with ocular neovascular diseases developed geographic atrophy (GA)^12^,^13^,^14^,^15^,^16^. Moreover, since patients with DR and other ocular neovascular disorders require long-term administration of anti-VEGF-A treatment, the development of GA may impose a serious problem. Thus, anti-apoptotic reagents that can protect retina from apoptosis and degeneration are highly desired.

VEGF-B was discovered in 1996 as a VEGF-A homologue with high sequence homology to VEGF-A^17^,^18^. Like VEGF-A, VEGF-B binds to VEGFR1 and NP1. However, unlike VEGF-A, VEGF-B does not play a significant role in inducing blood vessel growth or vascular permeability^19^. Instead, VEGF-B has been shown to be a potent neuroprotective factor and an inhibitor of apoptosis for different types of neurons^19^,^20^,^21^,^22^,^23^. Indeed, VEGF-B is highly expressed in different types of neural tissues, such as the brain^22^,^24^, retina^20^, and spinal cord^21^. However, it remains thus far unknown whether the expressions of VEGF-B and its receptors are regulated by hyperglycemia, and whether VEGF-B could be used to inhibit hyperglycemia - or anti-VEGF-A-induced retinal apoptosis. Notwithstanding, despite the many unanswered questions regarding the function of VEGF-B in the eye, drugs that can block VEGF-B are being used to treat patients with neovascular diseases^25^,^26^. It is therefore urgent to have a better understanding of the effect of VEGF-B in hyperglycemia and after VEGF-A inhibition.

To address the above questions, in this study, we used different animal models and cultured cells and investigated the effect of VEGF-B on retinal apoptosis and its expression under conditions of high glucose and VEGF-A inhibition. We found that in two retinal apoptosis models induced by diabetes or Macugen respectively, VEGF-B inhibited retinal apoptosis in different retinal layers. We also found that *in vitro*, the expressions of VEGF-B and its receptor VEGFR1 are upregulated by high glucose with concomitant activation of Akt and Erk. Thus, our data show that VEGF-B has a retinal protective effect and may potentially be used to treat retinal degeneration in different ocular diseases such as diabetic retinopathy and VEGF-A inhibition-induced geographic atrophy. Modulation of VEGF-B activity in the eye needs to be practiced with careful consideration.

## Results

### VEGF-B and its receptors are expressed in normal rat retinae and choroids

**Figure 1:**
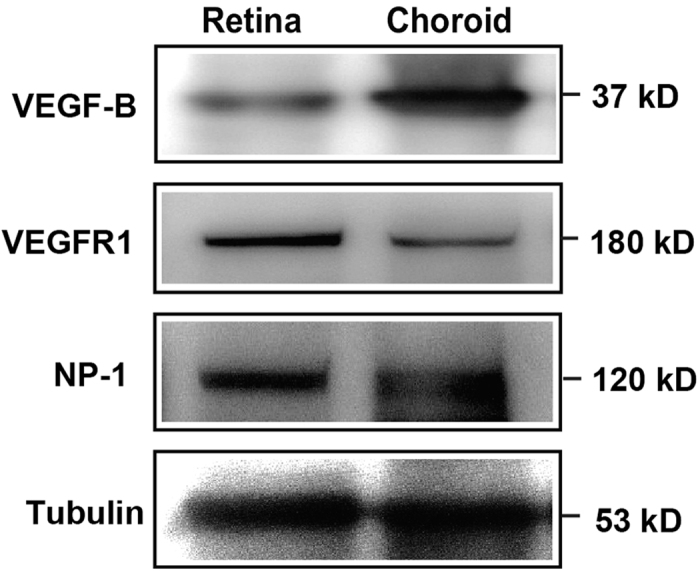
VEGF-B and its receptors are expressed in normal rat retinae and choroids. Western blot shows that VEGF-B, VEGFR1 and NP-1 are abundantly expressed in rat retinae and choroids using tubulin as an internal control.

The potential role of VEGF-B and its receptors in rat retina and choroid remains thus far unknown. To investigate this, we first examined the protein expression of VEGF-B and its receptors, VEGFR1 and NP1, in normal rat retinae and choroids. Western blot shows that VEGF-B, VEGFR1 and NP-1 are abundantly expressed in normal rat retinae and choroids (Fig. 1), implicating a role of VEGF-B in these tissues.

### Development of a rat diabetes model

**Figure 2:**
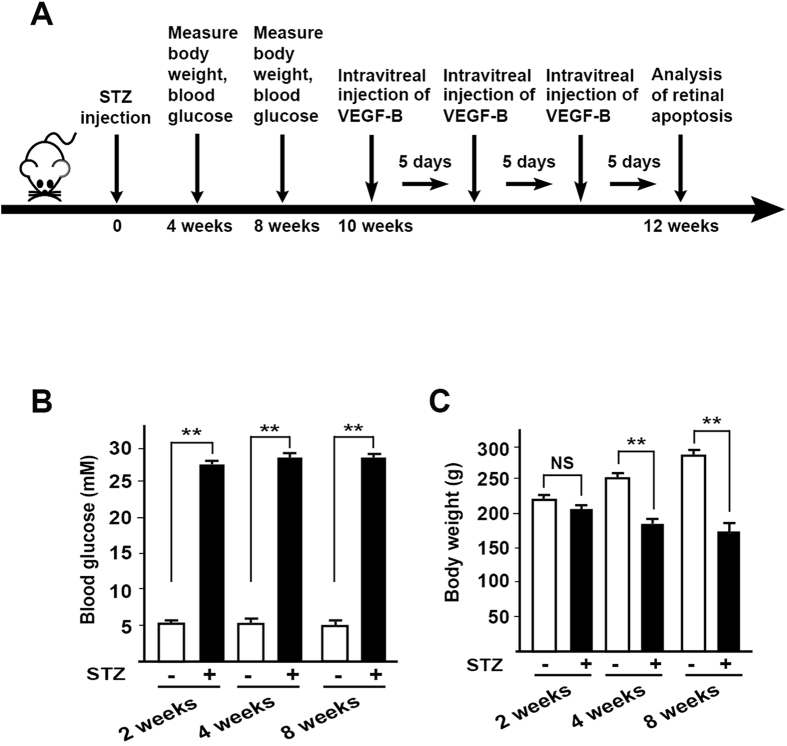
Development of a rat diabetes model. (**A**) A streptozotocin (STZ)-induced diabetes rat model was established by a single intraperitoneal injection of streptozotocin. Blood glucose levels of the rats were carefully monitored at different time points. (**B**) After STZ injection, blood glucose levels of the rats measured at different time points were significantly higher than those of the control group. (**C**) STZ treatment led to progressive loss of body weight in the streptozotocin-treated rats. Two-tailed Student’s t-tests were performed using independent samples. **P < 0.01.

It has been shown that VEGF-B is a survival factor for different types of neurons and can act as a neuroprotective factor^19^,^20^,^21^,^22^,^23^. However, it remains unknown whether VEGF-B could inhibit retinal apoptosis during hyperglycemia-induced retinal degeneration. To address this, we employed a streptozotocin (STZ)-induced diabetes model in rats, in which diabetes is induced by a single intraperitoneal injection of streptozotocin (STZ, Fig. 2A) and high glucose exposure. The blood glucose levels in these rats were carefully monitored to ensure the successful development of diabetes (Fig. 2A). Indeed, after STZ injection, the blood glucose levels measured at different time points were significantly higher than those of the control group (Fig. 2B, n = 16, *P* < 0.01). STZ treatment also resulted in progressive loss of body weight in the rats (Fig. 2C, n = 16, *P* < 0.01). These data thus show the successful development of a rat diabetes model.

### VEGF-B inhibits hyperglycemia-induced retinal apoptosis

**Figure 3:**
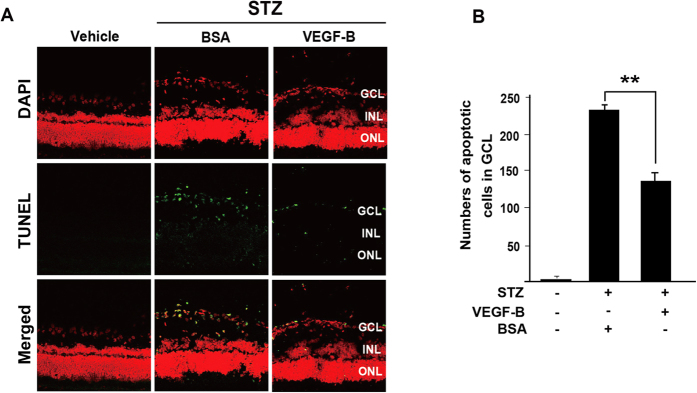
VEGF-B inhibits retinal apoptosis in diabetic rats. (**A**) TUNEL staining (green) detected apoptosis in rat retinae two months after STZ injection. Intravitreal injection of VEGF-B (500 ng/eye) significantly decreased the number of TUNEL^+^ cells compared with that of BSA-treated group. (**B**) VEGF-B treatment decreased the number of apoptotic cells by nearly 50% compared with that of BSA-treated rat retinae. Two-tailed Student’s t-tests were performed using independent samples. **P < 0.01.

Two months after STZ injection in the STZ-induced diabetic rats, terminal deoxynucleotidyl transferase dUTP nick end labeling (TUNEL) staining showed apoptosis in the rat retinae mainly in the retinal ganglion cell layer (GCL, Fig. 3A, left, green). Starting from the tenth week after STZ injection, intravitreal injection of VEGF-B (500 ng/eye) was performed every five days for fifteen days, after which the eyes were subjected to apoptosis analysis. TUNEL staining showed that VEGF-B treatment significantly decreased the number of TUNEL^+^ cells by nearly 50% compared with that of BSA-treated group (Fig. 3A,B, n = 10, *P* < 0.01). Thus, VEGF-B protected retinal cells from apoptosis in hyperglycemic retinae in rats.

### VEGF-B inhibits Macugen-induced retinal apoptosis

**Figure 4:**
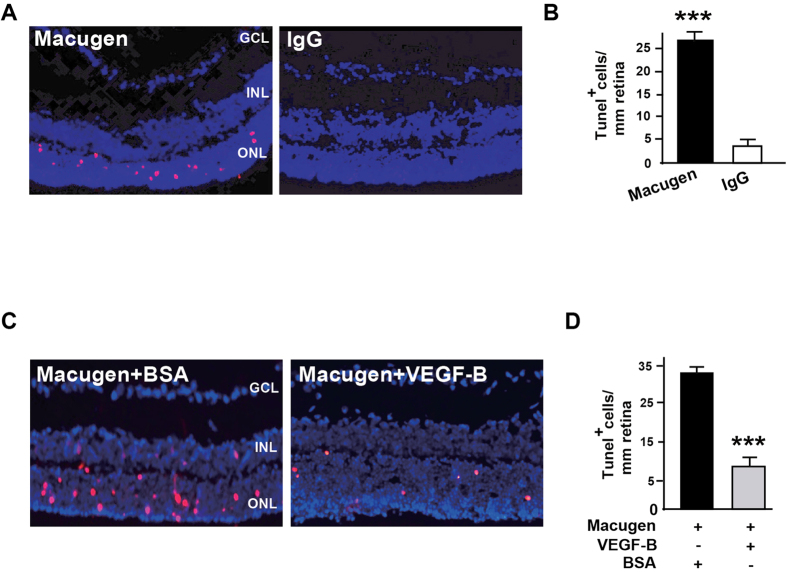
VEGF-B inhibits Macugen-induced retinal apoptosis. (**A**,**B**) TUNEL staining (pink) showed that intravitreal injection of Macugen resulted in apoptosis in the outer nuclear layer (ONL) of mouse retinae. (**C**,**D**) Co-injection of VEGF-B with Macugen markedly reduced the number of apoptotic cells in the mouse retinae. Two-tailed Student’s t-tests were performed using independent samples. ***P < 0.001.

It has been shown that inhibition of VEGF-A by intravitreal injection of Macugen (pegaptanib sodium) caused apoptosis in retinal photoreceptor cells in rabbits^27^. However, it remains unknown whether this is also true in mice, and, if yes, whether VEGF-B could protect retinae from Macugen-induced apoptosis. To answer this, we intravitreally injected Macugen with or without VEGF-B into mouse eyes and exmained retinal apoptosis seven days after injection. TUNEL staining (pink) showed that intravitreal injection of Macugen in mice resulted in apoptosis mainly in the outer nuclear layer (ONL) of the retinae (Fig. 4A,B, n = 8, *P* < 0.001). Importantly, co-injection of VEGF-B markedly decreased Macugen-induced apoptosis in the retinae (Fig. 4C,D, n = 8, *P* < 0.001). Thus, VEGF-B inhibits Macugen-induced retinal apoptosis and may have a therapeutic potential in rescuing VEGF-A inhibition-induced retinal degeneration.

### VEGF-B expression is upregulated by high glucose

**Figure 5:**
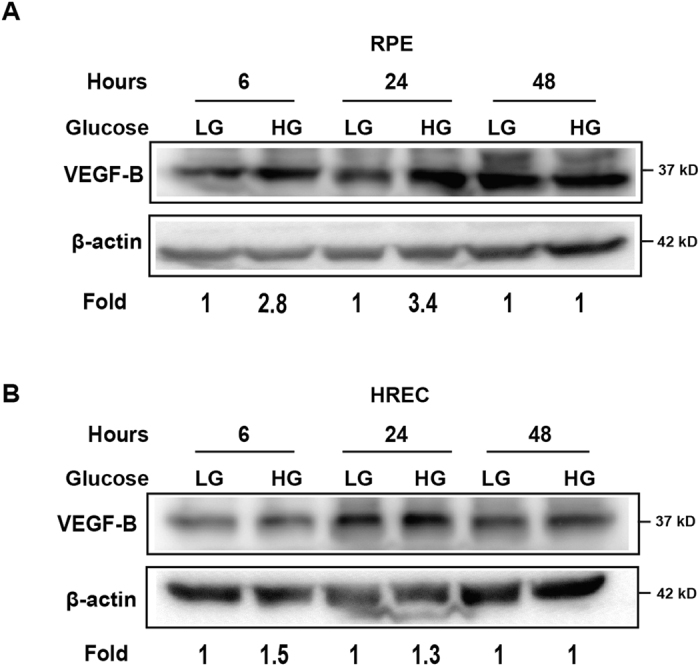
High glucose upregulates VEGF-B expression. (**A**) Western blot shows that VEGF-B protein level was upregulated in RPEs by high glucose as early as six hours after treatment and sustained until twenty-four hours in cultured human retinal pigment epithelial (RPE) cells. (**B**) Western blot shows that in human retinal endothelial cells (HRECs), VEGF-B protein level was slightly upregulated by high glucose at different time points tested. The cropped blots are used and full-length blots are included in the supplementary information.

It is thus far unknown whether the expression of VEGF-B is regulated by glucose level. We subsequently investigated whether the expression of VEGF-B is regulated by high glucose using cultured human RPE cells and human retinal endothelial cells (HRECs), which are known to be major sources of VEGFs in the retina^28^. Western blot showed that VEGF-B protein level was upregulated in RPEs by high glucose (HG) as early as six hours after treatment and sustained until twenty-four hours (Fig. 5A). In HRECs, VEGF-B protein level was slightly upregulated by high glucose at different time points tested (Fig. 5B). These data thus implicate a role of VEGF-B in diabetic retinae.

### The expressions of the receptors for VEGF-B are upregulated by high glucose

**Figure 6:**
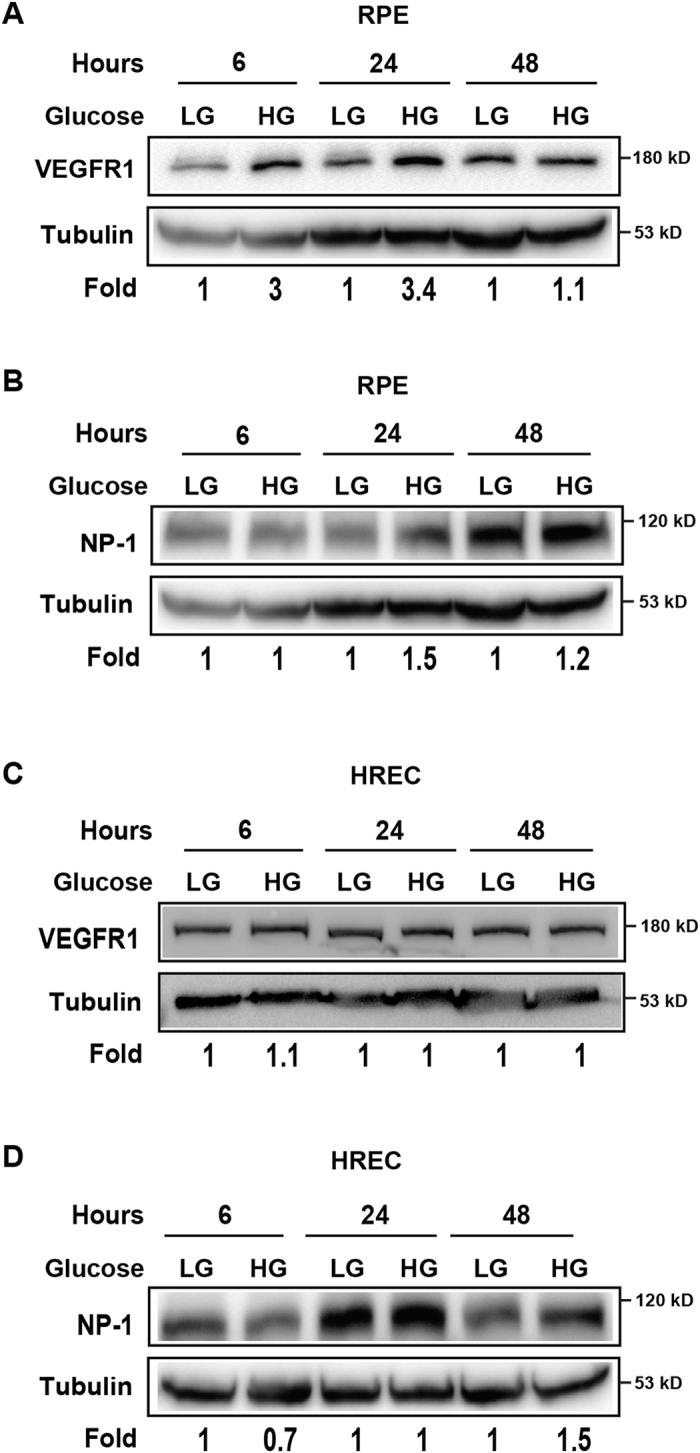
High glucose upregulates VEGFR1 and NP-1 expression. (**A**,**B**) Western blots show that the expressions of VEGFR1 and NP1 were upregulated by high glucose in RPE cells at different time points. (**C**,**D**) Western blots show that high glucose did not significantly change VEGFR1 or NP1 expression in HRECs at different time points tested. The cropped blots are used and full-length blots are included in the supplementary information.

VEGF-B binds to VEGFR1 and NP-1^29^,^30^. It is thus far unclear whether their expression is modulated by high glucose. In cultured RPEs and HRECs, Western blot shows that VEGFR1 and NP1 expressions were upregulated by high glucose in RPE cells at different time points (Fig. 6A,B). In HRECs, however, VEGFR1 expression did not show significant change at different time points tested (Fig. 6C). NP-1 expression was upregulated forty-eight hours after high glucose treatment (Fig. 6D). These findings suggest possible roles of VEGFR1 and NP1 in diabetic retinae. They also indicate that RPEs may be a major cellular target of VEGF-B under a high glucose condition.

### High glucose leads to activation of Akt, Erk but not Src

**Figure 7:**
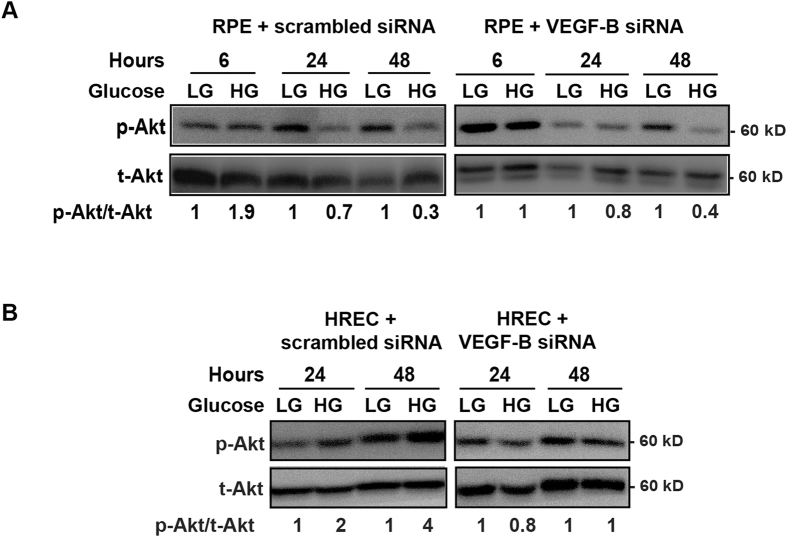
Activation of Akt by high glucose. (**A**,**B**) Western blots show that in cultured human RPE cells and HRECs, high glucose led to Akt activation six hours after treatment. Western blots also show that VEGF-B knockdown by siRNA abolished the high-glucose-induced Akt activation in RPE and HRECs. The cropped images of the blots are presented here and the full-images of the blots are included in the supplementary information.

**Figure 8:**
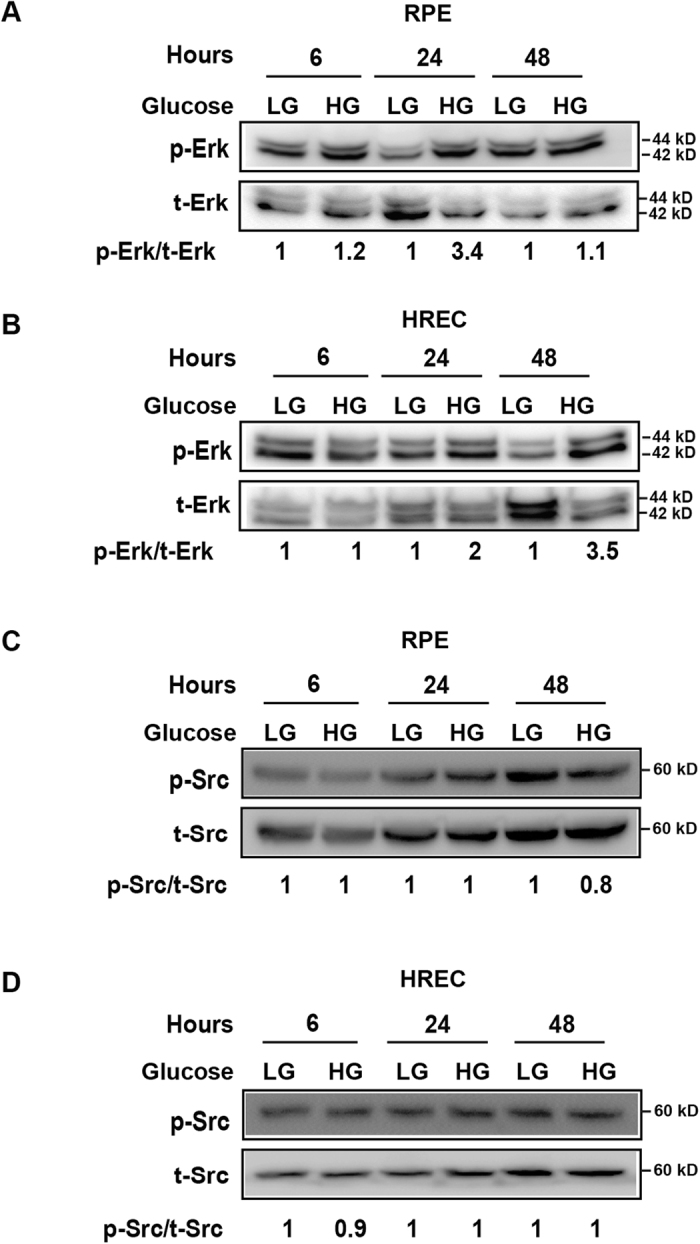
Activation of Erk but not Src by high glucose. (**A**,**B**) Western blots show that in cultured RPE cells and HRECs, high glucose resulted in Erk activation twenty-four hours after treatment. (**C**,**D**) Src activation was not changed by high glucose at different time points in RPE cells or HRECs as shown by Western blots. The cropped images of the blots are presented and the full-images of the blots are included in the supplementary information.

It has been shown that high glucose affects different signaling pathways^31^. However, it is poorly known whether high glucose affects signaling pathways in retinal pigmented epithelial cells as compared with retinal vascular endothelial cells. To address this, we employed cultured human RPE cells and HRECs and investigated Akt, Erk and Src activation under normal and high glucose conditions. We found that in both RPE cells and HRECs, high glucose led to Akt activation six hours after treatment (Fig. 7A) and VEGF-B knockdown by siRNA abolished Akt activation. In addition, Erk activation was induced by a high glucose twenty-four hours after treatment (Fig. 8A,B). By contrast, Src activation was not changed by high glucose at different time points in RPE cells or HRECs (Fig. 8C,D). These data thus suggest that Akt and Erk activation may play a role in relationship to VEGF-B function under conditions of high glucose.

## Discussion

Retinal degeneration by apoptosis can lead to blindness if uncontrolled and is a common characteristic of numerous ocular disorders, such as diabetic retinopathy, anti-VEGF-A therapy induced geographic atrophy^12^,^13^,^14^,^15^,^16^, and age-related macular degeneration. Currently, there is no efficacious treatment for such retinal degenerative diseases. In this study, we found that both streptozotocin (STZ)-induced diabetes and Macugen intravitreal injection resulted in retinal apoptosis. Importantly, VEGF-B treatment markedly reduced retinal apoptosis in both models *in vivo*. We further reveal that *in vitro,* VEGF-B and its receptors, VEGFR1 and NP1, are abundantly expressed in rat retinae and choroids and are upregulated by high glucose with concomitant Akt and Erk activation. Our data thus demonstrate a potential therapeutic usage of VEGF-B in treating retinal degenerative diseases. Inhibition of VEGF-B for other therapeutic purposes thus needs to be practiced with careful consideration.

Diabetes is a life threatening disease with a rapidly soaring incidence worldwide^1^. Diabetic retinopathy is a common complication of diabetes and the leading cause of blindness in working population in developed countries. Diabetic retinopathy is traditionally considered as a microvascular disease. However, mounting evidence has shown that it is also a neurodegenerative disease with progressive apoptosis of retinal neurons^1^. Although anti-VEGF-A drugs have been used to treat DR and other different types of ocular neovascular diseases and have proved to be a success, recent reports have shown that anti-VEGF treatment can lead to the development of geographic atrophy^12^,^13^,^14^,^15^,^16^,^32^. Indeed, VEGF-A has been shown to play an important role in the maintenance and function of retina and choriod 7. In patients, different clinical trials of anti-VEGF-A therapy have identified a significant number of patients with retinal atrophy after anti-VEGF therapy.

Retinal neuroprotection is a rapidly advancing field with great therapeutic potential^2^. Indeed, neuroprotective strategies that can rescue endangered cells from apoptotic cell death have been successful in both *in vitro* and *in vivo* experiments in halting or reversing damaged retinal neurons, thereby arresting the advancement of retinal degeneration^33^. Notably, most retinal degenerative pathologies are multifactorial with complex etiology. One advantage of neuroprotective treatment is that it may protect retinae from degeneration regardless of the multiple factors involved. Therefore, neuroprotection may prove to be a feasible general treatment for different retinal degenerative pathologies.

VEGF-B was initially discovered as a VEGF-A homologue^17^,^18^. However, it has little angiogenic effect under most conditions^19^. Instead, VEGF-B has been shown to be a potent neuroprotective factor and an inhibitor of apoptosis^19^,^20^,^21^,^22^,^23^,^34^. Even though both DR and GA involve complex and multi-etiological factors, retinal apoptosis is a common characteristic of both. VEGF-B therefore could be used to rescue retinal degeneration in both pathologies as a general treatment. In addition, apart from its neuronal survival effect, VEGF-B has also been shown to be a potent survival factor for multiple types of vascular cells, including vascular endothelial cells, pericytes, and smooth muscle cells^19^,^35^. Since blood vessel defects are critically involved in most degenerative diseases, it is possible that VEGF-B could also protect the vascular systems from degeneration in different degenerative pathologies, such as in hyperglycemia or VEGF-A deficiency.

In summary, using two different retinal degeneration rat and mouse models induced by high glucose and inhibition of VEGF-A with Macugen respectively, we show that VEGF-B treatment inhibited apoptosis in the retinae in both models. The expressions of VEGF-B and its receptors are upregulated by high glucose with Akt and Erk activation. These data demonstrate that VEGF-B may have an important therapeutic value in treating ocular degenerative diseases by preserving endangered retinal cells from apoptosis.

## Materials and Methods

### Streptozotocin-induced rat diabetes model

All animal experiments were approved by the Animal Use and Care Committee of Zhongshan Ophthalmic Center at the Sun Yat-Sen University. All animals were handled in accordance with the approved guideline. Male Brown-Norway rats (6–8 weeks) were purchased from Vital River (Beijing, China). For diabetes induction, the rats were fasted overnight prior to diabetes induction. The rats were anesthetized and diabetes was induced by a single intraperitoneal injection of streptozotocin (STZ) (50 mg/kg, S0130, Sigma). Age-matched control rats were injected with the vehicle (0.1 mol/L citrate buffered saline, pH 4.5). After injection, the rats were supplied with food and water with 10% sucrose. Blood glucose concentration of the rats was measured using a glucometer (Roche) according to the manufacturer’s instructions. The rats were diagnosed with diabetes when their blood glucose concentration exceeded 16 mmol/L one week after STZ injection. Their bodyweight and blood glucose concentration were subsequently measured at 1, 2, 3 and 4 months after STZ injection. All rats were housed individually and provided with food and water ad libitum in an air-conditioned room under 12-hour light/dark cycle. Ten weeks after STZ injection, intravitreal injection of VEGF-B (PeproTech) or BSA was performed (500 ng/eye) every five days and for three times. Five days after the last injection of VEGF-B/BSA, the rats were euthanized and eyes harvested for analysis. All rats were intraperitoneally anesthetized with chloral hydrate (350 mg/kg).

### Macugen-induced mouse retinal apoptosis model

All animal experiments were approved by the Animal Use and Care Committee of Zhongshan Ophthalmic Center at the Sun Yat-Sen University or the National Eye Institute, National Institutes of Health (NIH), USA. All animals were handled in accordance with the approved guideline. Intravitreal injection of Macugen (100 ng/eye, a kind gift from Dr. Wai Wong at the National Eye Institute, NIH, USA) was performed using 6–8 weeks old C57Bl6 mice. Seven days after injection, the eyes were harvested for apoptosis analysis.

### Terminal transferase dUTP nick end labeling (TUNEL)

The eyes of the rats or mice were harvested and embedded in optimal cutting temperature (OCT) compound (Leica) with the 9 and 12 o’clock positions of the corneal limbus facing two marker lines in the embedding container. The whole eye was cryosectioned throughout in parallel with the 12 o’clock meridian, and all the sections collected. The section with the optic nerve visible on it was used for analysis to ensure that similar positions of the eyes are analyzed. The sections were fixed in 4% paraformaldehyde for 20 minutes, and then incubated in 1% Triton X-100 in 0.1% sodium citrate for 10 minutes at room temperature. The fixed sections were incubated with terminal deoxynucleotidyl transferase conjugated with fluorescein (Roche, Indianapolis, IN), which labels the free 3’OH DNA at 37 °C for 50 minutes. The sections were examined with a microscope (Axio Vision, Zeiss) using a 20X objective. TUNEL-positive cells on each section were counted.

### Cell culture

Primary human retinal endothelial cells (HREC, Angio-Proteomie) were cultured in endothelial cell media (ECM, ScienCell) containing 5% fetal bovine serum, 1% penicillin/streptromycin (30-002-CI, Corning) with endothelial growth supplement (ScienCell). Human retinal pigment epithelial cells (ARPE-19, ATCC CRL-2302) were cultured in DMEM (Corning) containing 10% fetal bovine serum (FCS500, Excell Bio) and 1% penicillin/streptromycin. Normal medium (low glucose) contained 5 mM D-glucose (G5767, Sigma). High-glucose medium contained 40 mM D-glucose. The HRECs or RPE cells were cultured in normal or high-glucose medium for 6, 24 and 48 hours respectively, and then subjected to experiments.

### siRNA transfection

The RPE cells and HRECs were plated into 6-cm culture dishes and maintained to about 70–80% confluent. VEGF-B siRNA or control siRNA (RiboBio, Guangzhou, China) were transfected into the cells using Lipofectamine RNAiMAX (Invitrogen) according to the manufacturer’s protocols. After 48 hours, the transfection medium was replaced with low glucose or high glucose medium. The cells were continuously incubated at 37 °C with 5% CO_2_ and the cells were harvested for protein analysis at different time points.

### Western blot

For Western blots using tissues, the eyeballs of the rats were harvest, and the retinae and choroids dissected. The retinae and choroids were then lysed in 100 μl RIPA buffer (50 mM Tris-HCl, 150 mM NaCl, 1% NP-40, 0.5% Sodium deoxycholate, and 0.1% SDS) with protease/phosphatase inhibitors (Thermo Scientific). The protein concentrations of the tissue lysates were determined using a protein assay kit (Bio-Rad). For Western blots using cultured cells, the cells were plated in 60-mm tissue culture plates and cultured under different conditions until the cells reached 80–90% confluence. The cells were then washed twice with cold PBS, lysed in RIPA buffer with protease inhibitor and phosphatase inhibitor cocktails (Thermo Scientific). The cell lysates were centrifuged and protein concentrations determined using a protein assay kit (Bio-Rad). The cell lysates were subjected to Western blot using SDS-PAGE gel and transferred to PVDF (162–0177, BIO-RAD) membranes. The membranes were incubated with different antibodies, including anti-phospho (Tyr416)-Src (#2101, Cell Signaling), anti-Src (#2123, Cell Signaling), anti-phospho (Ser473)-Akt (#4060, Cell Signaling), anti-Akt (#4691, Cell Signaling), anti-phospho (Thr202/Tyr204)-Erk1/2 (#4370, Cell Signaling), anti-Erk1/2 (#4695, Cell Signaling), anti-VEGFR1 (ab32152, Abcam), anti-NP-1 (ab81321, Abcam), and anti-VEGF-B (sc-80442, Santa Cruz). Immunoreactivity was detected using an HRP-conjugated secondary antibody and visualized using enhanced chemiluminescence (ECL, Thermo Scientific). For Western blot analysis, the band intensities were determined with ImageJ program (NIH, Bethesda, MD) and normalized to internal controls (tubulin or β-actin) or total protein.

### Statistics

Two-tailed Student’s t-test was used for statistical analysis between two groups. Statistical analyses of the mean and variance were performed using an Excel program. Difference was considered statistically significant when *P* < 0.05. Data are represented as mean ± s.e.m. of the number of the determinations.

## Additional Information

**How to cite this article**: Huang, D. *et al.* VEGF-B inhibits hyperglycemia- and Macugen-induced retinal apoptosis. *Sci. Rep.* **6**, 26059; doi: 10.1038/srep26059 (2016).

## Supplementary Material

Supplementary Information
