# Supplementary material for: VEGF-B inhibits hyperglycemia- and Macugen-induced retinal apoptosis
Source: Sci Rep. 2016 May 18;6:26059. doi: 10.1038/srep26059 (PMC4870690; doi:10.1038/srep26059)
Supplement: Supplementary Information [file srep26059-s1.pdf]

Supplementary Information

VEGF-B inhibits hyperglycemia- and Macugen-induced retinal apoptosis

Delong Huang[1, 2#], Chen Zhao[3#], Rong Ju[2], Anil Kumar[2], Geng Tian[1], Lijuan Huang[2], Lei Zheng[1, 2], Xianglin Li[4], Lixian Liu[2], Shasha Wang[2], Xiangrong Ren[2], Zhimin Ye[2], Wei Chen[2], Liying Xing[2], Qishan Chen[2], Zhiqin Gao[4], Jia Mi[1], Zhongshu Tang[2], Bin Wang[5*], Shuping Zhang[1*], Chunsik Lee[2*], Xuri Li[2, 1*]

[1]Center for Medical and Pharmaceutical Research, Binzhou Medical University, Yantai, Shandong, 264003, P. R. China; [2]State Key Laboratory of Ophthalmology, Zhongshan Ophthalmic Center, Sun Yat-sen University, Guangzhou 510060, P. R. China; [3]Department of Ophthalmology, The First Affiliated Hospital of Nanjing Medical University and State Key Laboratory of Reproductive Medicine, Nanjing Medical University, Nanjing 210029, P. R. China; [4]Medical Imaging Institute, Shandong Province Characteristical Key Subject, Medical Imaging and Nuclear Medicine, Binzhou Medical University, Yantai, 264003 P. R. China; [5]Department of Cell Biology, Weifang Medical University, Weifang, 261053 P. R. China;

Equal first authors

* Corresponding authors

For communication: lixr6@mail.sysu.edu.cn

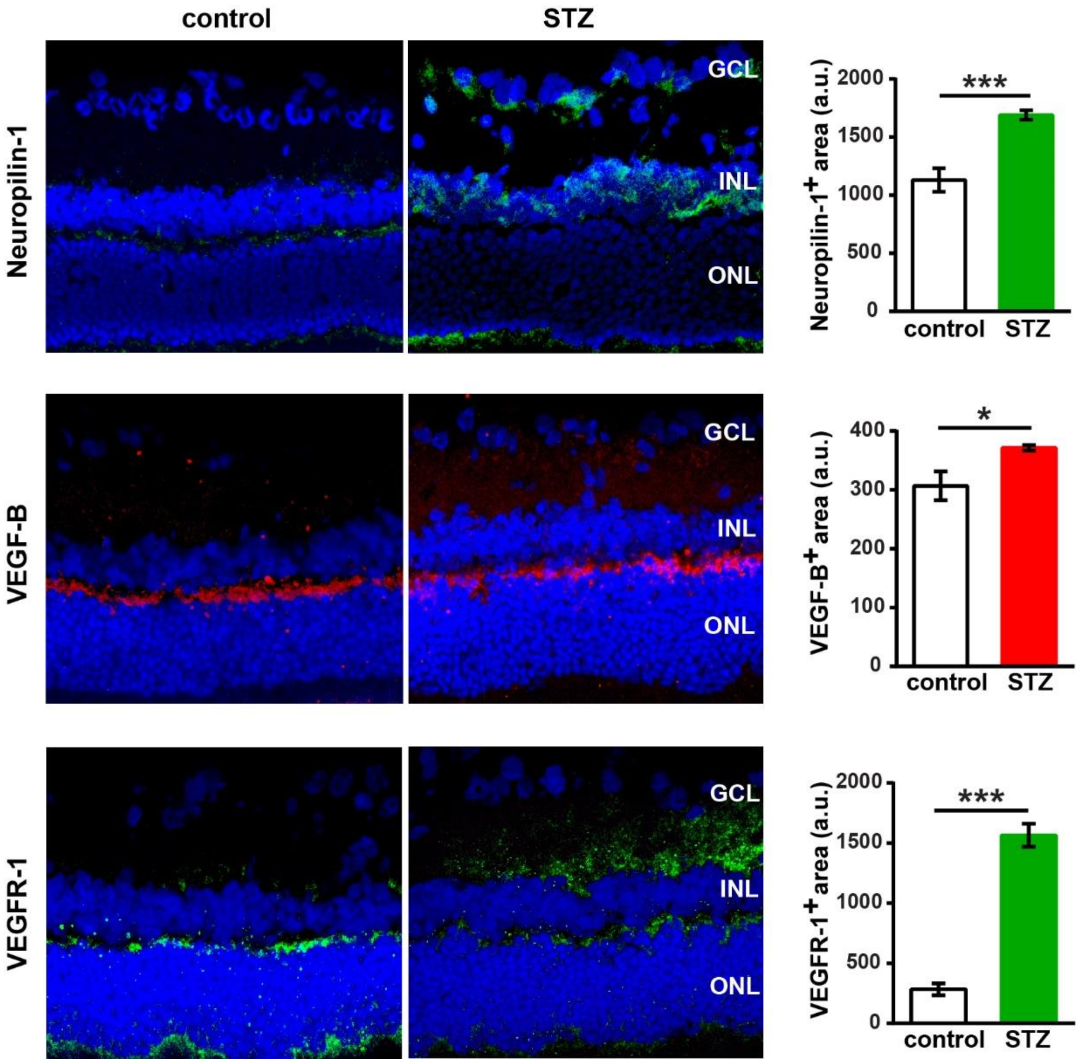

Supplementary Figure 1. NP1, VEGF-B, and VEGFR-1 are highly expressed in the retinae of STZ-induced diabetic rats.

Immunofluorescence staining of retinae from STZ-induced diabetic rats show that NP1, VEGF-B, and VEGFR-1 are highly expressed in the retinae of STZ-induced diabetic rats.

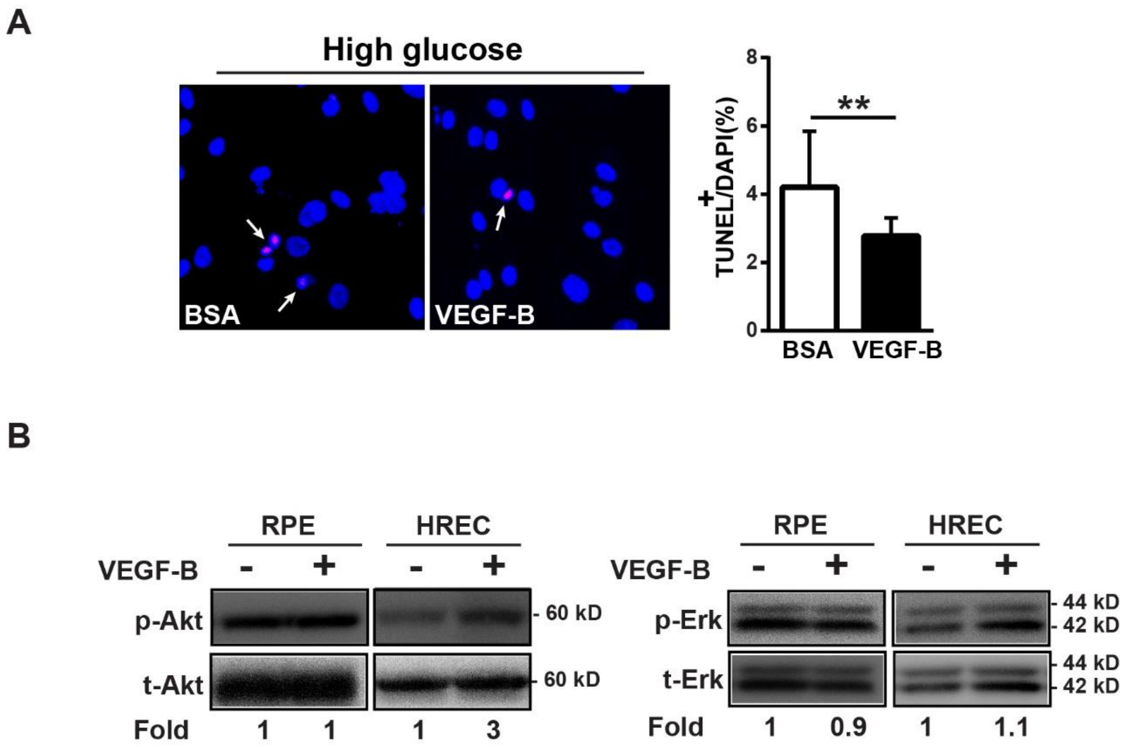

Supplementary Figure 2. VEGF-B inhibited apoptosis in HRECs and promoted Akt activation.

(**A**) TUNEL staining (red with white arrows) detected apoptosis in high-glucose-treated HRECs. VEGF-B (100 ng/ml) treatment significantly decreased the number of $TUNEL^{+}$ cells in HRECs as compared with those of BSA-treated ones. (**B**) Western blots show that VEGF-B induced phosphorylation of Akt but not Erk in HRECs.

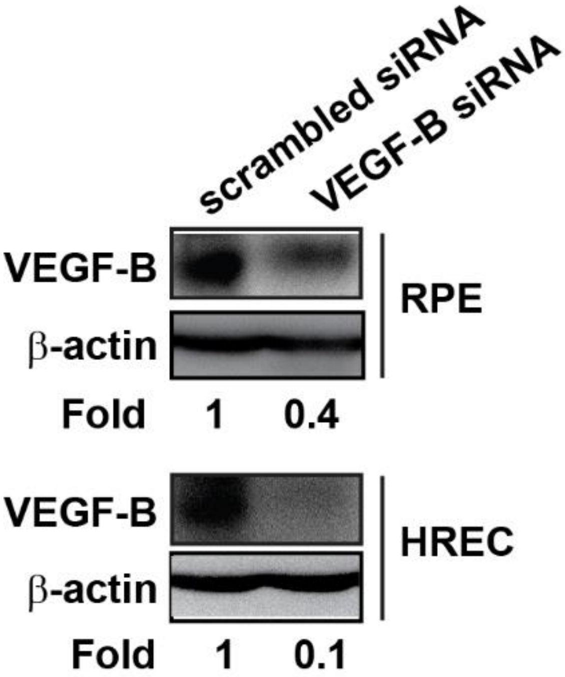

Supplementary Figure 3. VEGF-B knockdown by siRNA decreased VEGF-B expression.

RPE cells and HRECs were treated with VEGF-B siRNA and VEGF-B protein levels were decreased 48 hours after treatment as shown by Western blot.

(A)

Fig. 5A - RPE cells

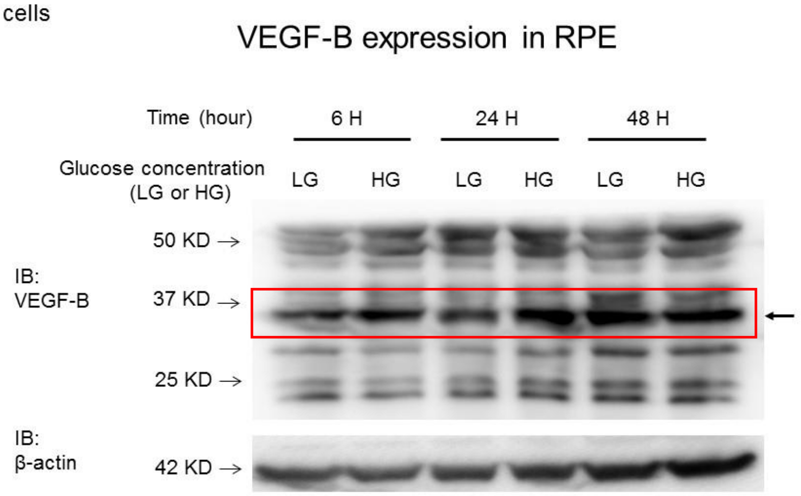

(B)

Fig. 5B - HREC

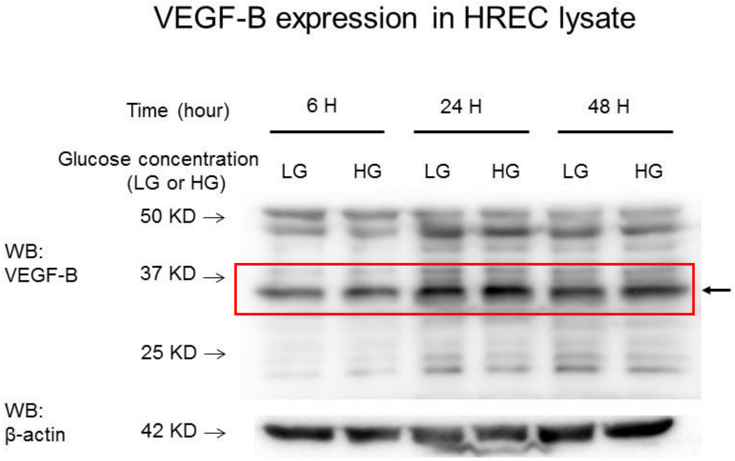

(C)

Fig. 6A - RPE cells

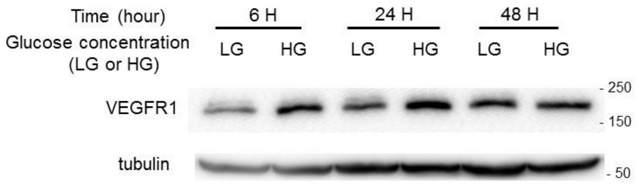

(D)

Fig. 6B – RPE cells

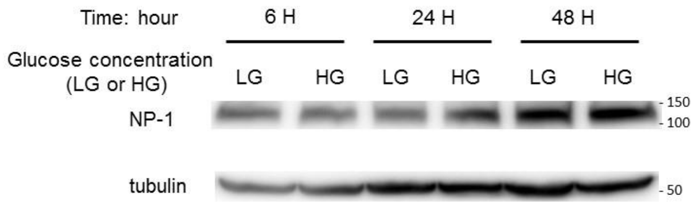

(E)

Fig. 6C – HREC

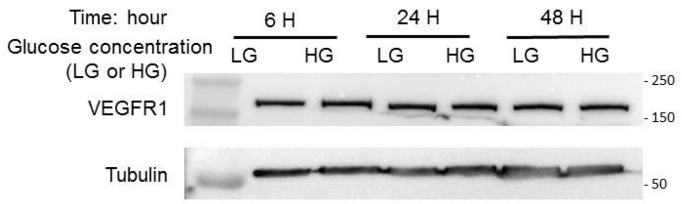

(F)

Fig. 6D – HREC

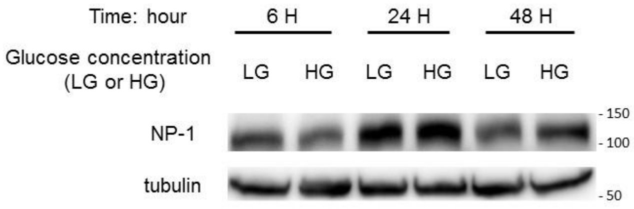

(G)

Fig. 7A & B

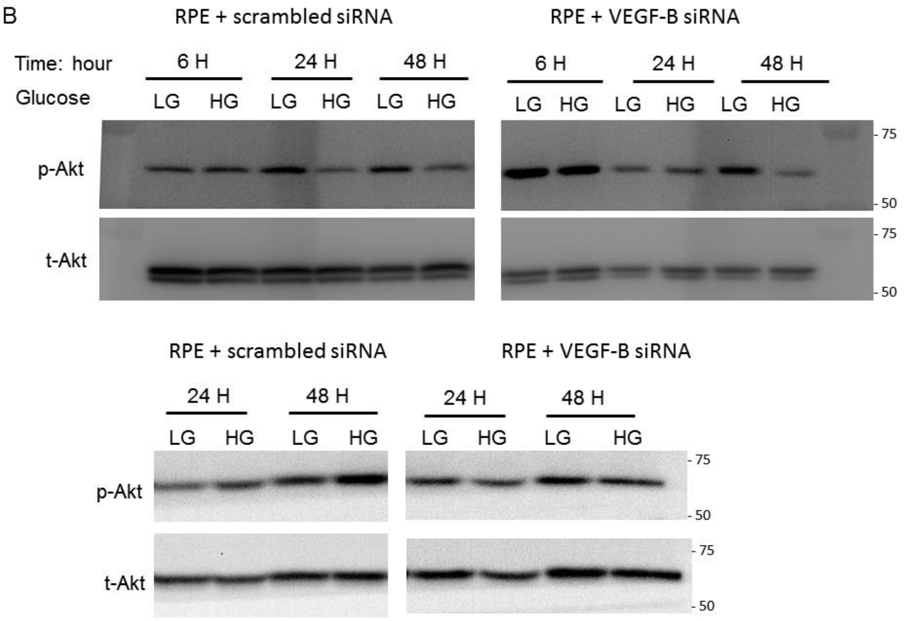

(H)

Fig. 8A – RPE cells

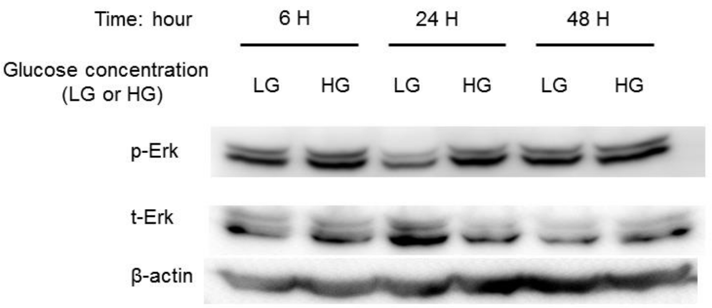

(I)

Fig. 8B - HREC

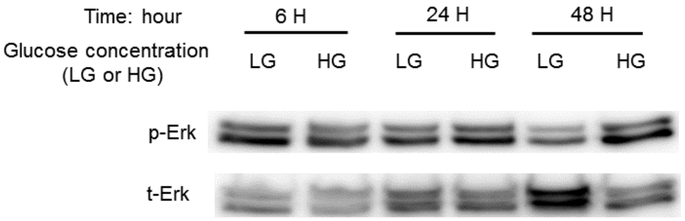

(J)

Fig. 8C – RPE cells

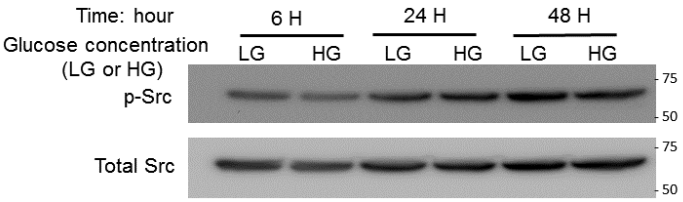

(K)

Fig. 8D – HREC

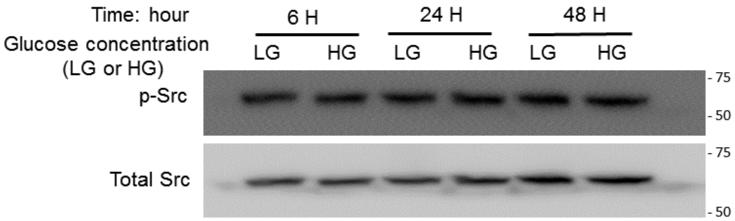

Supplementary Figure 4. Full length images of immunoblots.

The membranes were initially cut according to the molecular weight size of proteins. (A) Fig. 5A. (B) Fig. 5B. (C) Fig. 6A. (D) Fig. 6B. (E) Fig. 6C. (F) Fig. 6D. (G) Fig. 7A and B. (H) Fig. 8A. (I) Fig. 8B. (J) Fig. 8C. (K) Fig. 8D.
